# Aquatic therapy improves self-reported sleep quality in fibromyalgia patients: a systematic review and meta-analysis

**DOI:** 10.1007/s11325-023-02933-x

**Published:** 2023-10-17

**Authors:** Cristina Bravo, Francesc Rubí-Carnacea, Iolanda Colomo, Manuel Sánchez-de-la-Torre, Helena Fernández-Lago, Carolina Climent-Sanz

**Affiliations:** 1 Department of Nursing and Physiotherapy, University of Lleida, Montserrat Roig St. n2 P.C, 25198 Lleida, Spain; 2 Health Care Research Group, GRECS, Biomedical Research Institute of Lleida, Lleida, Spain; 3 Grup d’Estudis Societat, Salut, Educació i Cultura, GESEC, University of Lleida, Lleida, Spain; 4 Group of Salut&Genesis, Lleida Institute for Biomedical Research Dr. Pifarré Foundation, 25198 Lleida, Spain; 5 Group of Precision Medicine in Chronic Diseases, Hospital Arnau de Vilanova-Santa Maria, IRBLleida, Lleida, Spain; 6 Centro de Investigación Biomédica en Red de Enfermedades Respiratorias (CIBERES), Madrid, Spain

**Keywords:** Fibromyalgia, Hydrotherapy, Meta-analysis, Pain, Sleep quality

## Abstract

**Background:**

This systematic review and meta-analysis aimed to evaluate the effectiveness of aquatic therapy on pain, sleep quality, psychological symptoms, quality of life, and health status in people diagnosed with fibromyalgia.

**Methods:**

We searched PubMed, CINAHL, The Cochrane Library, PEDro and Scopus databases. Articles were eligible if they were randomised controlled trials (RCTs) analysing the effects of aquatic therapy in adult people diagnosed with fibromyalgia, and published by October of 2022 in English or Spanish. The Cochrane Risk of Bias tool was employed to conduct the methodological quality assessment of the encompassed studies, and the overall quality of evidence for each comparison was determined using the GRADE approach.

**Results:**

Of 375 articles found, 22 met the inclusion criteria. Forest plot analysis of Pittsburgh sleep quality index at short- and mid-term follow-up showed a trend in favour of aquatic therapy, although not statistically significant, with weighted mean difference (WMD) = -1.71 (95% CI: -4.17 to -0.75, *p* = 0.17). Heterogeneity was substantial (χ^2^ = 8.74, df = 5 (*p* < 0.000001; *I*^2^ = 95%). Relating the pain outcome by fibromyalgia impact questionnaire (FIQ) short term showed a trend in favour of the aquatic therapy group with WMD = −5.04 (95% CI: − 9.26 to − 0.82, p =  = 0.02) with heterogeneity *χ*^2^ = 11.07, df = 4 (*p* = 0.03; *I*^2^ = 64%). Great heterogeneity was found between trials in medium term.

**Conclusion:**

This systematic review and meta-analysis demonstrated the effectiveness of aquatic therapy as an adjunct treatment to usual care in people suffering from fibromyalgia. Aquatic therapeutic exercise improves the symptomats of sleep quality, pain, and quality of life of adults with fibromyalgia. Further research on long-term outcomes may contribute to the currently available evidence.

**Supplementary Information:**

The online version contains supplementary material available at 10.1007/s11325-023-02933-x.

## Introduction

Fibromyalgia (FM) is a chronic condition characterized by chronic widespread pain and is acknowledged as a significant contributor to disability [1, 2]. Clinical manifestations encompass a broad spectrum of chronic symptoms including chronic widespread pain, fatigue, sleep disturbances, and psychological disorders [1]. The global prevalence of FM stands at 2.1%, with a higher incidence among women (4.3%) compared to men (0.95%), resulting in a global gender ratio of 4:1. Europe exhibits a higher prevalence of 2.3%, with the highest incidence recorded in countries such as Turkey, Italy, Portugal, Germany and Spain [3, 4].

The standard clinical approach to managing FM involves a combination of pharmacological and non-pharmacological treatments. Among the latter, various interventions are applied, including aerobic exercises, flexibility exercises, strength training, stretching and body awareness therapies [1].

Aquatic therapy or aquatic exercise refers to the application of aquatic properties to design a therapy program to improve patient functionality. Water is an environment that, due to its properties, provides the opportunity for the patient to undertake global activity and to apply physiotherapy that, outside the water, could not possibly be done [5]. The four main physical principles of water are buoyancy, resistance, hydrostatic pressure and thermal conduction [5]. The decrease in pain is attributed to a conjunction of factors, i.e. exercise, warm water and buoyancy that activate mechanoreceptors and thermoreceptors. Immersion in warm water increases blood flow and therefore oxygen in the blood, eliminating catabolites and reducing the level of IL-8 and noradrenaline, responsible for activating nociceptors [5]. This sensory and motor hyperstimulation blocks nociceptors and reduces the patient’s pain. The physiological effects provided by aquatic therapeutic exercise results from immersion in warm water (26–32 °C) [6, 7], which reduces the activity of the sympathetic nervous system and can decrease inflammation and pain perception in subjects with musculoskeletal disorders. Benefits are also obtained through hydrostatic pressure, as compressive stresses on the joints are reduced and allow for functional exercise with gravitational discharge. This property allows for more intense exercise, strength, and range of motion with less cardiovascular stress [7]. However, one high-quality review incorporating 10 trials on hydrotherapy and spa therapy provided little evidence to suggest superiority over the comparator intervention [1].

In recent decades, aquatic therapy studies have been conducted suggesting that the physical benefits of water and the effects of exercise are effective in improving the symptoms of musculoskeletal diseases such as osteoarthritis, FM and rheumatoid arthritis, mainly in terms of pain [7]. The effectiveness of aquatic therapy has been assessed in a previous review published in 2013 [8] showing beneficial effects on physical fitness, wellness, and symptoms associated with FM. A study by Choy et al [9] indicated that sleep dysfunction may induce fibromyalgia-like symptoms and may have bidirectional roles in the pathophysiology of fibromyalgia. The concept of sleep quality is defined as an individual’s self-satisfaction with all aspects of the sleep experience that can be measured by the following variables: sleep efficiency, sleep latency, wake after sleep onset and sleep architecture measures [10]. A study by Theadom et al [11] and a systematic review [12] indicate that between 70 and 90% of people with FM report poor sleep quality and that exercise could ameliorate that disturbance. This systematic review and meta-analysis aimed to evaluate the effectiveness of aquatic therapy on sleep quality as a main outcome, and secondarily the outcomes of pain, quality of life, health status, and psychological symptoms, in people diagnosed with FM [12].

## Methods

The study entailed a systematic review of randomised controlled trials, adhering to the PRISMA (Preferred Reporting Items for Systematic Reviews and Meta-Analyses) guidelines [13]. This systematic review and meta-analysis was registered at PROSPERO with CRD42020201621. Only randomised controlled trials investigating the use of aquatic therapy exercise in the treatment of FM were considered. The articles were selected based on the following criteria: (a) studies involving adult participants diagnosed with FM by the International Classification of Diseases with the code M79.0 such as rheumatism [2]; (b) participants receiving treatment through a single aquatic therapy exercise method, compared to a placebo, control intervention or standard care, using a randomised controlled trial (RCT); (c) studies reporting any of the following outcomes regarding sleep, pain, quality of life, health status, and psychological symptoms measured by Pittsburgh sleep quality index (PSQI), fibromyalgia impact questionnaire (FIQ), visual analog scale (VAS) and short form 36 (SF-36) and (d) written in either the English or Spanish language.

The authors conducted searches in PubMed, CINAHL, The Cochrane Library, PEDro and Scopus. The search strategy was carried out based on the PRESS guidelines recommendations [14]. We combined Mesh, entry and free text terms such as “fibromyalgia”, “fibromyalgia syndrome”, “aquatic therapy” and “hydrotherapy”. The filter “clinical trial” was also used. The search was run on 30th October 2022. The references of the included studies were also reviewed to identify any additional studies. A manual search was conducted by cross-referencing with the studies selected in the earlier research.

The reports located via electronic searches were imported and checked for duplicates. Following the removal of duplicates and in accordance with the eligibility criteria, two independent reviewers (CB and IC) assessed publications retrieved from the databases based on their titles and abstracts in the initial phase, and the full texts in the subsequent phase. Any discrepancies were re-evaluated, and consensus was reached through discussion. If needed, a third author (HFL) reviewed the data to ensure consensus.

After selecting the articles that met the eligibility criteria, the studies underwent a risk of bias assessment using Cochrane tools [15] which also included a peer-review process. The evaluation considered potential sources of bias such as sequence generation, allocation concealment, blinding of participants and personnel, blinding of outcome assessment, incomplete outcome data and selective reporting. In accordance with Cochrane guidelines, each item was categorized as “low risk”, “high risk” or “unclear risk” of bias.

The data extracted from the included studies encompassed: (a) participants’ characteristics, (b) details about the experimental and control groups, (c) relevant outcomes and outcomes measures, (d) study results, (e) tools used for assessing outcomes and (f) any reported adverse effects.

To assess the treatment effects, the data were presented as continuous outcome data, and mean differences (MD) with 95% confidence intervals (CIs) were computed for instances where differences between-group in the mean from baseline were documented. Data analysis was conducted using Review Manager 5.4 software. Given the variability in aquatic therapies and the varying number of participants across studies, a random-effects model was employed to estimate the true effect of aquatic interventions.

In our study, we employed the *χ*^2^ test and the *I*^2^ statistic to assess heterogeneity [16]. The *χ*^2^ test measures the deviation of observed effect sizes from the underlying overall effect. A low *p* value indicates significant heterogeneity in intervention effects, with a significance threshold set at *p* < 0.05. The *I*^2^ statistic, as defined by Higgins [16], quantifies heterogeneity as a percentage: (a) 0–40% suggests negligible heterogeneity, (b) 30–60% implies represent moderate; (c) 50–90% suggests substantial heterogeneity and d) 75–100% indicates considerable heterogeneity. The importance of the *I*^2^ value depends on (a) magnitude and direction of effects and (b) strength of evidence for heterogeneity. When pooling data, we selected the MD and utilized forest plots to compare results across studies. To assess publication bias, we inspected funnel plots [17].

We assessed the overall quality of the evidence for each outcome. To accomplish this, we used a GRADE approach, as recommended by the Cochrane Back Review Group [18].

The quality of the evidence on a primary outcome was evaluated across five main domains: study design limitations (risk of bias assessment), inconsistency (lack of uniformity in bias assessment estimates), indirectness (inability to generalize) and imprecision (inadequate patient numbers or wide confidence intervals) of results and publication bias (likelihood of selective publication of trials and outcomes) across all studies examining that specific outcome. The evidence is categorized into four levels: (a) high-quality evidence: at least 75% of the RCTs exhibit consistent findings, lack limitations in study design, present direct and precise data and demonstrate no known or suspected publication biases; (b) moderate quality: when one of the domains is unmet; (c) low quality: when two of the domains are unmet; (d) very low quality: three of the domains are unmet.

## Results

### Studies’ selection

**Fig. 1:**
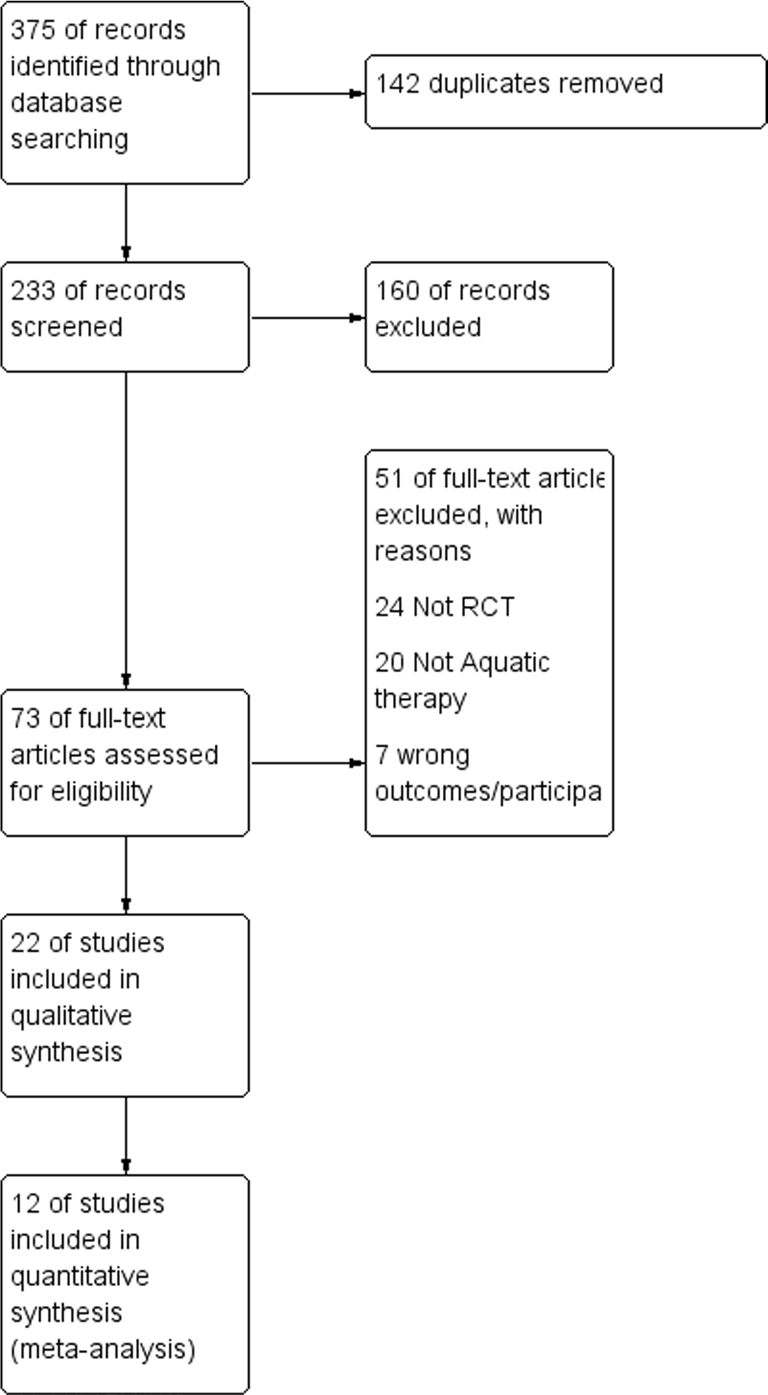
Flow diagram for the identification, screening, eligibility and inclusion of studies

The initial electronic database query yielded a total of 375 studies. We conducted supplementary manual searches of reference lists and websites, but they did not yield any additional articles. After eliminating duplicates, non-English language articles, non-randomised clinical trials within aquatic therapy exercise interventions, 353 articles were excluded. A total of 22 studies [19–40] were included in systematic review and meta-analysis (Fig. 1).

### Studies characteristics

A total of 22 RCTs involving 1722 participants were included in the study. Twenty [19–23, 25–37, 39, 40] of these studies employed a double-arm design, while two [24, 38] utilized a triple-arm design, allocating patients to aquatic therapy or alternative treatment groups. Among the 1722 participants, ages ranged from 18 to 60, with a mean age of 42.5 ± 12.3. In all studies, FM was diagnosed using the criteria of the American College of Rheumatology (ACR). The duration of the interventions ranged from 11 to 20 weeks.

For articles published by the same authors, participants were considered only once. The characteristics of the included studies can be examined in Table 1.

**Table 1:** Characteristics and results of the included studies

Authors	Participants	Intervention group versus control	Relevant outcomes	Results	Measurement tools	Adverse effects
Acosta-Gallego et al. (2018)^20^	*n* = 73 ♀ (30–59 yr). M = 48.2±6.8 No follow-up	Twice a wk during 20 wk - Pool based of standard physical rehabilitation intervention program (SPRI-P) (*n* = 37): 10-min warmup, 25 min of main aerobic exercise and 10 min of stretching and relaxation - Land based of standard physical rehabilitation intervention program (SPRI-L) (*n* = 36)	For self-perceived pain and perceived fatigue, in the SPRI-P, there was a reduction in post-test regarding pre-test (M = 6.7 ± 1.8 vs 5.7 ± 2.1; *p* = 0.012), (M = 8.2 ± 2.8 vs 7.3 ± 2.4; *p* = 0.04). Depressive symptoms, there was a considerable improvement in both programs (*p* < 0.001)	Pool program is more effective, yielding a reduction in the overall impact of FM. However, both programs produce improvements in physical symptoms such as self-perceived pain, perceived fatigue and decrease depression.	VAS, FIQ, HAM-D, 6MWT	Not provided
Andrade et al. (2008)^22^	*n* = 46 ♀ (18–65 yr). M = 48.8 ± 9.9 (PG) M = 48.3 ± 8.9 (SG) No follow-up	- Pool group (PG) (*n* = 23): Consists: 10-min stretching, 40-min low-impact aerobic exercise and 10-min relaxation. - Sea group (SG) (*n* = 23): It received the same of pool group 60 min, 3 times a week for 12 weeks.	The sea group presented a most expressive improvement in BDI score in post-treatment (*F* = 2.418, *P* < 0.0001)	Aerobic exercises performed in sea water should be a safe and of low-impact therapeutic option in depression symptoms.	BORG perceived exertion scale, VAS, FIQ, SF-36, PSQI, BDI,	Muscle pain. Burn by Portuguese caravel
Andrade et al. (2019)^23^	*n* = 54 ♀ (30–60 yr) M = 48 ± 8 vs 47± 8 Follow-up at 16 and 32 weeks	16 wk. of intervention - Aquatic exercise (*n* **= **27): twice a wk 10-min warmup, 30-min aerobic exercise and 5-min cooldown - Control group(*n* **= **27)	Variable VAS fatigue [*F*(1.27) = 4.68; *p* = 0.03] was improved. Also BAI [*F*(1.27) = 4.26; *p* = 0.04] and components “functional capacity”, “pain” and “vitality” of SF-36.	16 wk of aquatic exercise was effective in promoting VO_2_ and improved clinical symptomatology as pain, well-being. After 16 wk of detraining period, there was a return near baseline.	VAS, FIQ, SF-36, BDI, BAI, PSQI, cycle ergometer test, PPT	Not provided
Assis et al. (2006)^23^	*n* = 60 ♀ (18–60 yr) M = 42.2 ± 10.1 (DWR) M = 44.0 ± 8.9 (LBE) No follow-up	- Deep water running (DWR) (*n* = 30) Consists a running movement with floating belt, with no contact with the bottom of the pool. Temperatures to 28 to 31 °C. - Land-based exercises (LBE) (*n* = 30) Both groups exercised for 60 min, 3 times a week for 15 weeks. Follow-up: 8 and 15 weeks	FIQ depression scores showed significant differences between groups aftes 8 weeks (P=0.028, 95% CI 0.179-3.621) and 15 weeks (P=0.025, 95% CI 0.214-3.719). Also, SF-36 role emotional improved in weeks 0 and 15 (*p* = 0.012) for the DWR group.	DWR may bring advantages regarding emotional aspects such as depression and emotional role.	VAS, PGA, SF-36, BDI, FIQ, HR, Peak VO_2_	Not provided
Britto et al. (2020)^24^	*n* = 33 ♀ (35–56 yr) M = 50.3 ± 6.1 (WG); M = 46.8 ± 10.8 (LG) No follow-up	- Water-based exercise (WG) (*n* = 16): 60 min that consists: warm-up 10 min, stretching 10 min, strengthening 30 min and relaxation 10 min. In water pool at 33 °C. - Land-based exercise (LG) (*n* = 17). It included warm-up, active stretching, strengthening and relaxation with intensities similar in WG. During 8 weeks, three times a week	Significant difference between groups for the functional capacity variable of FIQ (*p* = 0.015). Significant difference between groups in relation to flexibility (*p* = 0.001) with considered average to excellent in 12 (75%) participants in the WG	The water-based exercise program increased functional capacity and flexibility regarding land-based exercise.	FIQ, VAS, number of tender points (TPs), Wells bench sit and reach test	Not provided
Cedraschi et al. (2004)^25^	*n* = 164 ♀ (18–60 yr) M = 48.9 ± 9.7 (IG); 49.8 ± 9.8 (CG) Follow-up 6 months	12 sessions, twice a week for 6 weeks. - Intervention group (IC) (*n* = 84): consists: swimming pool session, relaxation, low-impact land-based exercises, activities of daily living and education sessions. - Waiting list control group (CG) (*n* = 80)	The IG showed a significant improvement in the PGWB “anxiety”, “vitality” and total scores (*p* < 0.05). Also, in FIQ total score, “fatigue”, “pain” and “depression” subscales (*p* < 0.05)	The Intervention group found that improvements were sustained for at least 6 months after the programme completion in factors such as fatigue, depression, anxiety and vitality.	PGWB, SF-36, FIQ, RPS	Not provided
De Medeiros et al. (2020)^26^	*n* = 42 ♀ (18–60yr) M = 50.7 ± 9.7 (AAEG); 45.5 ± 10.6 (MPG) No follow-up	Twice a week during 12 wk - Aquatic aerobic exercise group (AAEG) (*n* = 21): 5-min warm up, 30-min aerobic exercise and 5 min of cooldown - Mat Pilates group (MPG) (*n* = 21): 50 min	There were improvements in AAEG in PSQI MD = 2.7 (*p* = 0.02) and in PRCTS MD = 0.74 (*p* = 0.01). FIQ improved in both groups with mean difference (MD) of 0.91 (*p* = 0.002) and 1.6 (*p* = 0.001) AAEG vs MPG. VAS also improved with MD of 1.8 (*p* = 0.001) and 1.3 (*p* = 0.01). MPG showed improvement in SF-36 in FABQ-Phys MD = 4.9 (*p* = 0.005).	Mat pilates and aquatic aerobic exercises were effective after 12 wks in improving pain.	VAS, FIQ, SF-36, PSQI, PRCTS, FABQ	Not provided
Evcik et al. (2008)^27^	*n* = 62♀ + 1♂ (18–60 yr). M = 43.8 ± 7.7 (AEP) M = 42.8 ± 7.6 (HBEP) Follow-up at 12 and 24 weeks	15 sessions 3 a week for 5 weeks - Aquatic exercise program (AEP) (*n* = 33): consists of 20-min warming up, active range of motion and relaxation, followed with 35 min of aquatic exercises and 5 min with cooling down - Home-based exercise program (HBEP) (*n* = 30): consists of warming up, ROM, relaxation, aerobic, stretching and cooling down exercises.	VAS showed significant differences at follow-up 24 weeks in aquatic therapy group (*p* = 0.010).	Both groups have beneficial effects in the treatment of physiological well-being, quality of life and pain parameters. However, aquatic therapy seems to have more advantage in long-term pain management.	Algometer, VAS, FIQ, BDI,	Not provided
Fernandes et al. (2016)^28^	*n* = 75 ♀ (18–60 yr) M = 48.3 ± 8.9 (SG) M = 49.3 ± 9.2 (WG) No follow-up	Three weekly sessions during 12 wks - Swimming group (SG) (*n* = 39): 50 min of swimming with a heart rate at 11 beats under the anaerobic threshold - Walking group (WG) (*n* = 36): same duration and frequency of SG	Statistically significant differences were found in the within analysis with respect to the VAS, FIQ, SF-36 and TUGT (*p* < 0.001).	A swimming and walking program had similar and beneficial effects on pain, functional capacity and quality of life in patients with FM.	VAS, FIQ, SF-36, TUGT, Spiroergometric test, analgesic log	Not provided
Fernandes De Melo et al. (2006)^29^	*n* = 50 ♀ (30–60 yr) M = 48.9 ± 9.2 (HT) M = 46.6 ± 8.4 (CP) No follow-up	- Hidrotherapy group consists: warm up 5 min, stretching 6 min, aerobic exercises 30 min, relaxation 13 min - Conventional physiotherapy: infrared lamp 10 min, stretching 5 min, aerobic exercise 30 min, relaxation 10 min.	There were statistically significant differences in the HT group regarding TST (*p* < 0.01). Also, increased at least 1 h in TST regarding CP patients (*p* = 0.04) and TNT decreased significantly in HT (*p* < 0.05)	HT and CP are equally effective to improve QOL for FM patients, but HT is more effective than CP to improve TST and to decrease TNT	TST total sleep time, TNT total nap time, QOL, SF-36	Not provided
Fonseca et al. (2019)^30^	*n* = 46 ♀ (25–60 yr) M = 53.7 ± 10.40 vs 54.4 ± 11.18 No follow-up	11 wk of intervention - Aquatic exercise (*n* = 27): once a wk 60-min intensity moderate (50%) 5-min warm up, 15-min stretching, 30-min active exercise and 10-min relaxation and cooldown - Inter-relational School of FM (ISF) group (*n* = 19) once a wk 60 min, training cope strategies, nutrition, symptoms and treatment of FM.	The two-way mixed ANOVA demonstrated that there was an effect on functional capacity [*F*(1.82, 80.41) = 31.99; *p* ≤ 0.01], in sleep [*F*(1.71, 75.08) = 14.42; *p* ≤ 0.01] and anxiety [*F*(1.34, 58.34) = 18.98; *p* ≤ 0.01] from the post-test to the other measurements in both groups. The interaction between group and time was for the functional capacity of FIQ [*F*(1.82, 80.41) = 31.99; n2 = 0.125; *p* ≤ 0.05]	The findings do not allow to affirm that one intervention is superior to the other. Between-group analyses suggest that ISF may have a greater effect only on the impact of FM on participants’ lives than aquatic physiotherapy.	McGill Pain Questionnaire, PFS-R, FIQ, BAI, BDI, PSQI	Not provided
Gowans et al. (2004)^31^	*n* = 16 ♀ + 2♂ M = 47.3 ± 2.4 Follow-up: 6 and 12 months	- Exercise group (EX): 3 sessions per week for 23 weeks. It consists of 10-min stretching, 20-min aerobic exercise. The first 6 weeks were conducted in warm pool. At 7 weeks consists in 2 walking classes and 1 pool class by week. HR 60–75% of age-adjusted maximum - Control group: waiting list.	Walk distances and BDI total scores were significantly at the end of 23 weeks of exercise and at 6- and 12- months follow-up (*p* < 0.05).	Physical function and depression can be improved up to 12 months following 23 weeks of supervised exercise classes	BDI, 6MWT, FIQ, ASES, STAI, Mental Health Inventory, Tender point	Not provided
Gusi et al. (2006)^32^	*n* = 34 ♀ M = 51 ± 10 (EX) M = 51 ± 9 (CG) Follow-up at 6 months	-Exercise group (EX) (*n* = 17): 1 hour 3 times per week for 12 weeks. It consists of 10-min warming up , 10-min aerobic exercises at 65–75% maximal HR, 20-min mobility, 10-min aerobic exercises and 10-min cooling down - Control group (CG) (*n* = 17): waiting list	Pain, quality of life subscales of mobility, selfcare, pain and anxiety were improved statistical significantly at 12-week follow-up. Also, at 24-week follow-up, quality of life self-care subscale and anxiety significantly showed differences.	The exercise relieved pain and improved quality of life and muscle strength in the lower limbs. The quality of life was maintained in the long-term.	Strength muscle measurement, EQ-5D, VAS	Not provided
Kurt et al. (2016)^33^	*n* = 109 ♀ (19–63yr) (37.2 ± 12.5) M = 38.1 ± 10.1(G1); M = 35.1 ± 11.6 (G2); M = 41.9 ± 12.8 (G3) Follow-up at 3 months	-Group 1 balneotherapy (*n* = 37): 20 min 5 days a week at 42 ± 1 °C. 15 sessions in total -Group 2 balneotherapy + exercise (*n* = 36) it started with 25 min and was extended to 35 min. It consists in stretching, strengthening and relaxation exercises with a heart rate 60 to 70% -Group 3 exercise (*n* = 36)	Groups 1 and 2 had better scores than group 3 in terms of FIQ, PSQI and TMS at post-treatment and third-month control (*p* < 0.05). BDS in groups 2 and 3 showed significant differences than group 1 at post-treatment and third-month control (*p* < 0.05).	The combined treatment of balneotherapy and exercise was more effective in relation to pain, quality of sleep, impact of disease and depression.	FIQ, PSQI, BDS, Total myalgic score (TMS)	Not provided
Latorre Román et al. (2015)^34^	*n* = 369 ♀ M = 50.3 ± 8.8 (CG); 51.7 ± 9.5 (EG)7 No follow-up	Three weekly sessions during 18 wks -Intervention: 2 aquatic sessions and 1 land session by week (*n* = 20): 5- min warm up, 40-min muscular strengthening and balance and 5 min of cooldown - Control group (*n* = 16)	After intervention, a significant reduction was observed in the FIQ (*p* = 0.042), the algometer scale (*p* = 0.008), positive points (*p* < 0.001) and VAS (*p* < 0.001) in group intervention.	A 18 wk of twice sessions in-water exercise and one session of on-land exercise reduces pain and disease impact and improves functional capacity.	VAS, FIQ, 30-second Chair Stand Test, 8-Food Up and Go Test, tork, Balance Stand test	Not provided
Lopez-Rodriguez et al. (2012)^35^	*n* = 39 (18–65 yr) M = 55.5 ± 7.7 (EG); M = 55.3 ± 7.5 (CG)	60 min twice a week during 12 weeks -Aquatic biodanza group (*n* = 19) consists: (a) stretching and warm up 10 min, (b) biodanza movements 40 min, (c) stretching 10 min. The water was 29 °C. - Stretching group (*n* = 20)	Intervention group showed FIQ score 52.16(16.18), VAS score 5.42 (2.19), BDI score 16 (7.39) and McGill score 28.68 (6.69) with *p* < 0.05	There were significant differences between groups, in pain, FM impact and depression after the treatment.	FIQ, McGill-Melzack questionnaire, VAS, Pressure algometry, BDI	Not provided
Lopez-Rodriguez et al. (2013)^36^	*n* = 59 ♀ (18–68 yr) M = 55.5 ± 7.8 (EG); M = 54.2 ± 7.2 (CG)	60 minutes twice a week during 12 weeks - Aquatic biodanza group (*n* = 29) consists: (a) flexibility and breathing 10 min, (b) biodanza movements 40 min, (c) stretching 10 min. The water was 29 °C. -Stretching group *n* = 30	Significant differences between groups on PSQI 7.59 (1.8), SAI score 38.79 (5.8), FIQ score 53.73 (18), VAS score 5.17 (2.1).	There were significant differences between groups in sleep quality, anxiety, pain and FM impact.	PSQI, SAI, CESDS, VAS, Algometry and McGill-Melzack questionnaire, FIQ	Not provided
Maindet et al. (2021)^37^	*n* = 218 (> 18 yr); 90.8% ♀ Age M = 49.8 ± 8.8 Follow-up at 6 months	3 wk of intervention - Intervention group (IG) *n* = 110: spa therapy - Control group (CG) *n* = 108: delayed spa therapy (after 6-month follow-up)	FIQ decrease in the intervention group at 6 months. Also, VAS (58.9 vs 53.5, *p* < .05). PCS-CF (29.4 vs 25.4, *p* < .001), HAD depression (10.5 vs 9.4, p < .05), Pichot’s Fatigue (25.2 vs 22.9, *p* < .05), SSSS (9.5 vs 9.0, *p* < .05) and Coping Scale Score (20.5 vs 19.9, *p* < .05)	Assessed spa therapy provides a long-term beneficial clinical effect for patients suffering from moderate to severe fibromyalgia	FIQ, EQ-5D-3L, PGA, IGA, VAS, PCS-CF, WPI score, SSSS, PSQI, Epworth Scale, Pichot Scale, HAD, Coping questionnaire, BAECKE questionnaire, BMI	Not provided
Saltskar Jentoft et al*.* (2001)^38^	*n* = 34 ♀ (20–60 yr) M = 42.9 ± 8.6 (PE) M = 39.4 ± 8.8 (LE) Follow-up at 46 months	20 wk of intervention - Pool-based exercise (PE) (*n* = 18): Norwegian aerobic fitness model lasted 60 min. It consists of body awareness, ergonomics, warm up, aerobic dance, cooling down, stretching, strengthening and relaxation exercises. - Land-based exercise (LE) (*n* = 16)	The LE group had improved their grip strength after 20 weeks compared with the PE group (*p* = 0.02)	Physical capacity can be increased by exercise, even when the exercise is performed in a warm water pool.	FIQ, VAS, Tender point, SES, cardiovascular capacity O_2_ uptake, grip strength, endurance time, walking time of 100m	Not provided
Sevimli et al*.* (2015)^39^	*n* = 75 ♀ (18–50 yr). M = 35.0 ±8.8 No follow-up	12 wk of intervention - Home-based isometric strength and stretching exercise program (ISSEP) (*n* = 25): 15 min daily -Gym-based aerobic exercise program (AEP) (*n* = 25). Twice a wk - Pool-based aquatic aerobic exercise program (AAEP) (*n* = 25): twice a wk	VAS and BDI values showed significant differences in their pre-and post-test values in all three groups. FIQ, 6MWT, mental and physical component of SF-36 between pre-and post-test showed significant differences.	AAEP and AEP are effective methods for FM. AAEP had additional effects on the SF-36 mental health and BDI. AAEP was also significantly more effective than AEP and ISSEP.	Tender points, VAS, FIQ, 6MWT, SF-36, BDI,	Not provided
Tomas-Carus et al*.* (2007)^40^	*n* = 34 ♀ (35–73 yr) M = 51 ± 10 (EG) M = 51 ± 9 (CG) No follow-up	- Pool-based exercise (EG) (*n* = 17): water at 33 °C 3 timer per wk for 12 wk. It consists of 10-min warming up, 10-min aerobic exercises at 60–65% maximal HR, 20-min mobility, 10-min aerobic exercises and 10-min cooling down. - Control group (CG) (*n* = 17): waiting list	Subscales of FIQ such as physical function (*p* = 0.016), well-being (*p* = 0.003), work capacity (*p* = 0.046), pain (*p* = 0.03), stiffness (*p* = 0.038), anxiety (*p* = 0.044) and depression (p = 0.046)	Physical exercise in warm water was an effective treatment to decrease the pain in fibromyalgia women	FIQ	Not provided
Tomas-Carus et al*.* (2008)^41^	*n* = 30 ♀ (35–73 yr). M = 50.7 ± 10.6 (EG) M = 50.9 ± 6.7 (CG)	-Pool-based exercise (EG) (*n* = 15): water at 33 °C 3 timer per wk for 8 wk. It consists of 10-min warming up, 10-min aerobic exercises at 60–65% maximal HR, 20-min mobility, 10-min aerobic exercises and 10-min cooling down - Control group (CG) (*n* = 17): waiting list	FIQ total scores showed differences between groups (*p* < 0.05). Also, subscales such as physical function, pain, stiffness, anxiety and depression (*p* < 0.05). Also, STAI showed differences between groups (*p* = 0.035). The physical function were improved with differences statistically such as maximal O^2^ uptake (*p* = 0.015),step stair-climbing and walking speed (*p* < 0.05)	Exercise led to long-term improvements in physical and mental health in patients with fibromyalgia.	Postural balance, SF-36	Not provided

*ASES*, Arthritis Self-Efficacy Scale; *BAI*, Beck’s Anxiety Symptoms; *BDI*, Beck’s Depression Inventory; *BDS*, Beck Depression Scale; *BMI*, body mass index; *CESDS*, Center for Epidemiologic Studies Depression; *CPET*, Cardiopulmonary Exercise Test; *EQ-5D-3L*, quality of life 5 dimension; *FABQ*, Fear Avoidance Beliefs Questionnaire; *FIQ*, Fibromyalgia Impact Questionnaire; *HADs*, Hospital Anxiety and Depression Scale; *HAM-D*, Hamilton Depression Scale; *IGA*, investigator global assessment; *MHI*, Mental Health Inventory; *6MWT*, 6-minute walk test; *PC-CF*, Pain Catastrophizing Scale (French); *PFS-R*, Piper Fatigue Scale-Revised; *PGA*, Patient Global Assessment; *PGWB*, Psychological General Well-Being; *PPT*, Pressure Pain Threshold; *PRCTS*, Catastrophic Thoughts on Pain Scale; *PSQI*: Pittsburgh Sleep Quality Index; *RPS*, Regional pain score; *SAI*, State Anxiety Inventory; *SES*, Self-Efficacy Scale; *STAI*, State-Trait Anxiety Inventory; *SF-36*, Short Form 36; *SSSS*, Symptom Severity Scale Score; *TMS*, Total Myalgic Score; *TUGT*, Time Up and Go Test; *VAS*, Visual Analogue Scale; *WPI*, Widespread Pain Index Score; wk: week

### Risk of bias and quality of evidence

**Fig. 2:**
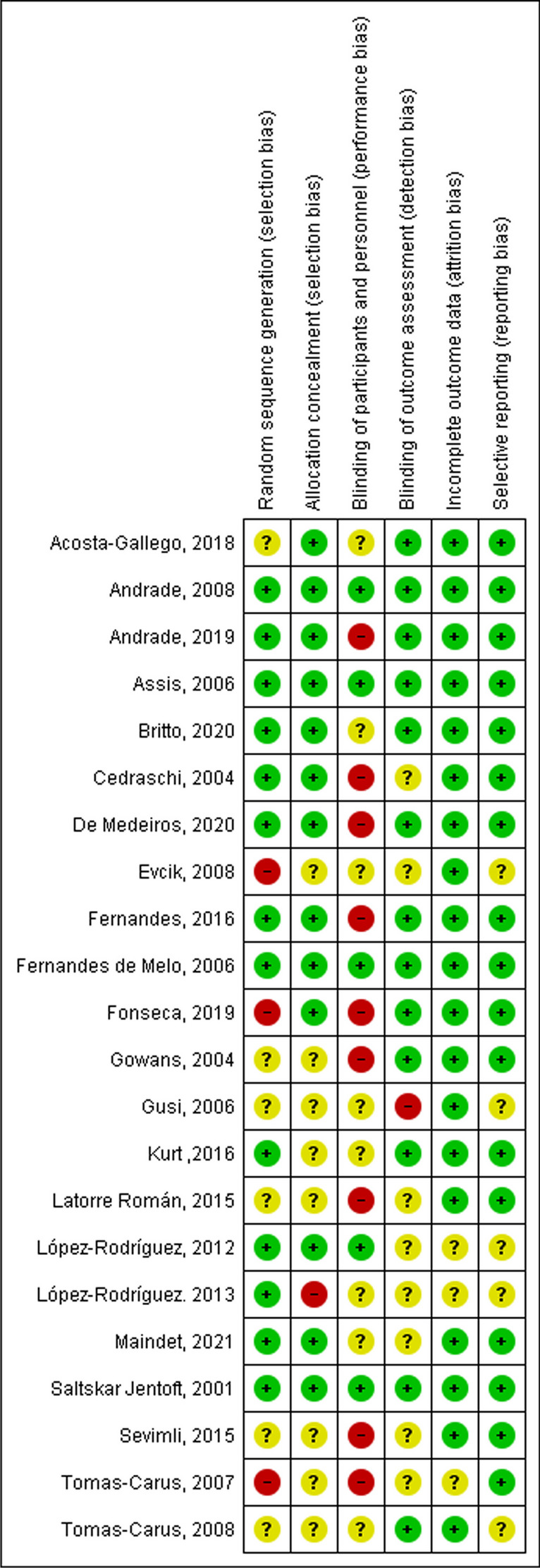
Risk of bias summary: review authors’ judgements about each risk of bias item for each included study

In relation to the risk of bias, the primary concern identified was performance bias, as both the participants and personnel were not effectively blinded to the specific intervention received by each participant. It can be argued that blinding is not feasible in this kind of study and that lack of blinding should not be considered a weakness. Furthermore, blinding of the outcome assessors and blinding of the patients to the hypothesis of the study are possible. Other important biases were sequence generation and allocation concealment bias, as most of the studies did not describe in sufficient detail how participants were randomized and allocated (see Fig. 2).

There is moderate quality of evidence as assessed with the GRADE approach that aquatic therapies are more effective than usual care in the treatment of fibromyalgia. Although the domains of inconsistency, indirectness and imprecision were assessed as adequate, the risk of bias as a limitation of study was a weakness of the methodological studies.

### Results of individual studies and synthesis of the results

We conducted a random-effects meta-analysis on the following outcome measures: PSQI, FIQ (short term, mid term and long term), VAS, and SF-36. Based on the results and observed heterogeneity, the outcomes that we reported in our publication include PSQI, FIQ mid term and FIQ short term, as well as VAS mid term.

### Sleep quality

The effects of aquatic therapy regarding to sleep quality were evaluated using the PSQI by four studies in a short-term follow-up [29, 32, 35, 36] and by two in short and mid-term follow-up [20, 25]. The most common instrument to measure the quality of sleep self-reported that it found is the Pittsburgh Sleep Quality Index in which it is possible to evaluate duration, sleep latency, frequency and severity present in the period of 1 month prior to the date [41]. PSQI was a reliable and valid instrument (*r* = 0.77, *p* ≤ 0.01) with the FIQ total score and with the mental and physical health summaries scores of the SF-36 [42]. However, Fernandes de Melo study [28] used total sleep time and total nap time as outcome to assess sleep quality.

A total of 6 studies with 231 participants by group provided available data of PSQI at pre- and post-treatment periods. Our analysis showed a trend in favour of aquatic therapy, with plot values of PSQI 1.71 points larger than the control group such as Mat Pilates, health education, balneotherapy or land stretching (weight mean difference or WMD = − 1.71, 95% CI: − 4.17 to − 0.75, *p* = 0.17; heterogeneity *χ*^2^ = 8.74, df = 5, *p* < 0.000001; *I*^2^: 95%). The observed outcome lacks statistical significance, and due consideration should be given to the heterogeneity among studies concerning the utilized aquatic therapy interventions and control groups. No significant bias was observed by the inspection of the funnel plot of outcomes analyzed (Figure S1). The sensitivity analyses were employed to assess the robustness of the results using the leave-one-out method. The analyses confirmed the stability of results for PSQI, FIQ short and mid-term and VAS mid-term (Table S1–S4).

### Important outcomes

**Fig. 3:**
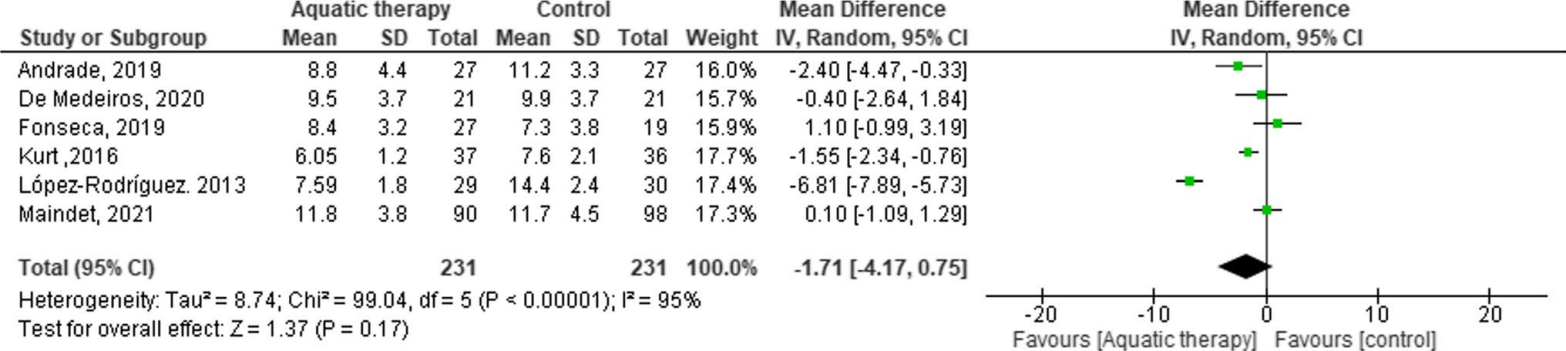
Forest plot of comparison: PSQI short and mid-term follow-up

**Fig. 4:**
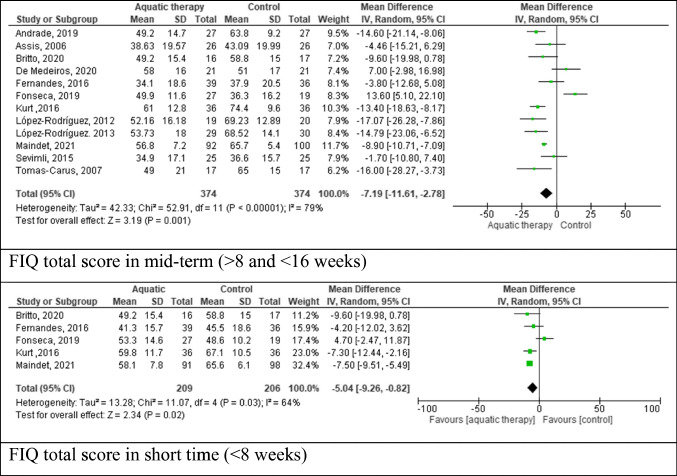
Forest plot of comparison: FIQ total score in mid-term (>8 and <16 weeks) and short time (<8 weeks)

**Fig. 5:**
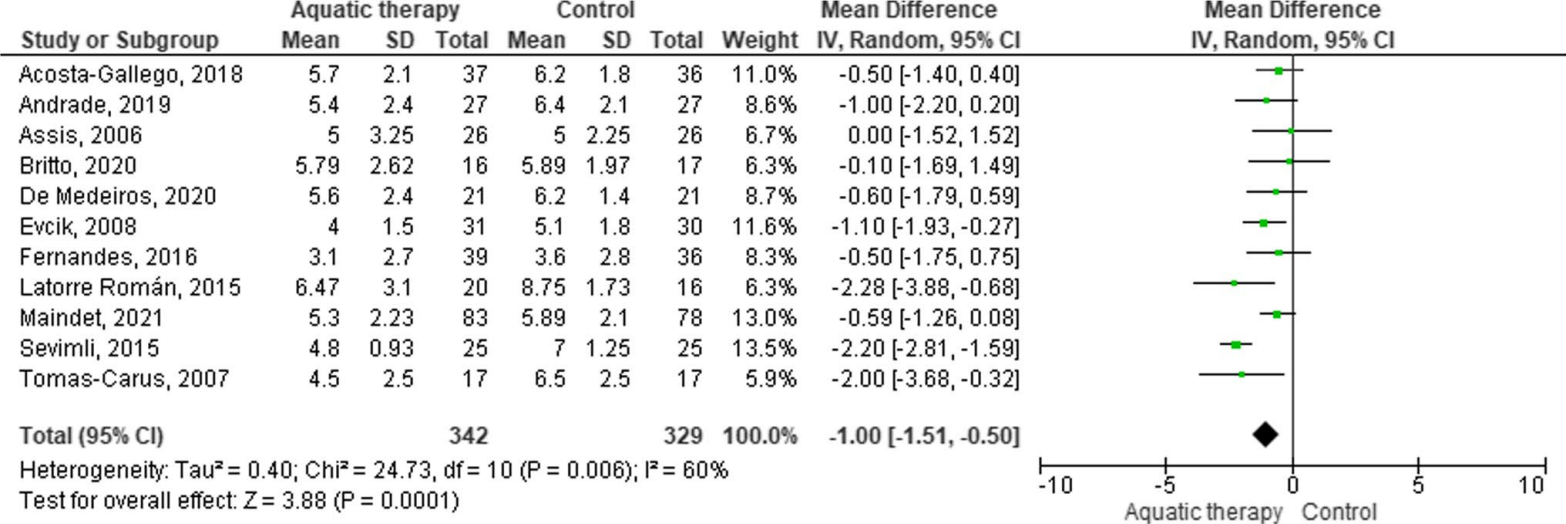
Forest plot of comparison: VAS mid-term follow-up

The FIQ scores were used to estimate summary effects. Based on the time of follow-up and FIQ score evaluation, two different subgroups were defined, namely mid-term (> 8 and < 16 weeks) and short time (< 8 weeks). In mid term, analysis showed a trend in favour of aquatic therapy with plot values of FIQ score of 3 points larger than the control group control (WMD = − 7.19. 95% CI: − 11.61 to − 2.78, *p* = 0.001); heterogeneity *χ*^2^ = 52.91, df = 11, *p* < 0.01, *I*^2^ = 79%). A total of 12 studies with 748 participants provided available data of FIQ at mid term at pre- and post-treatment periods. In the medium term, all the studies showed outcomes in favour of aquatic therapy, except for the Fonseca [29] and De Medeiros [25] studies that compared aquatic therapy with an educational intervention and mat Pilates. In long term, two of six studies [20, 33] compared aquatic therapy to a control group (without treatment), Acosta-Gallego [19] compared aquatic therapy with physical intervention on land and Maindet [36] study compared spa treatment with delay treatment. Due to the variety in the control group intervention in the studies included in the medium-term analysis, a substantial degree of heterogeneity was detected. However, in the long-term analysis, the interventions were similar, and the heterogeneity was null, but the results are not statistically significant. At short time, the results’ trend is in favour of aquatic therapy with a FIQ score of 2 points larger than the control group (WMD = − 5.04 [95% CI: − 9.26 to − 0.82]; comparison: *p* = 0.02); heterogeneity *χ*^2^ = 11.07, df = 4 [*p* = 0.03]; *I*^2^ = 64%). A total of 5 studies with 415 participants provided available data of FIQ at short term at pre- and post- treatment periods. In the short term, five studies collected data; our analysis showed a positive trend in favour of aquatic therapy in four studies [23, 27, 32, 36], but the Fonseca [29] study showed a trend in favour of the Inter-relational School of FM (Figs. 3, 4 and 5).

Regarding the VAS Score evaluation, a forest plot was created for the medium-term intervention. A total of 11 studies with 671 participants provided available data of VAS at mid-term at pre- and post-treatment periods. The plot value trend was in favour of aquatic therapy in comparison with control (WMD = − 1.00 [95% CI: − 1.51 to − 0.50]; comparison: *p* = 0.006); *χ*^2^ = 24.73, df = 10 [*p* = 0.0001]; *I*^2^ = 60%). In that comparison, the control interventions were control without treatment [20, 33], aerobic exercise [19, 22, 23, 26, 27, 38, 39], delay spa treatment [36] and mat Pilates [25].

The assessment tools used were PSQI in 6 articles, FIQ was used in 12 studies and VAS in 15 articles, SF-36 in 9 articles and BDI in 8 articles. The FIQ is considered one of the most sensitive tools to assess FM evolution over time and is a key endpoint in clinical trials aiming at evaluating an individual’s responsiveness to different intervention models [43]. The range of possible total scores is 0 to 10 on 10 items for the measurement of physical function and severity of symptoms.

### Effects on physical outcomes

The main outcome related to physical symptoms of patients with fibromyalgia is pain. The most common tools that have been used are the FIQ, VAS and SF-36. However, one study [29] also used the McGill Pain Questionnaire and Pressure Pain (PPT) and Widespread Pain Index Score [36]. Other physical capacity outcomes were measured using the 30-s chair stand test, the 8-foot up and go test [33] and Baecke physical activity questionnaire [36].

In the studies by Andrade [20] and Fonseca [29], the perception of pain was diminished but not with a significant result between groups (*p* > 0.05), although the pre-test and post-test measures of Andrade showed differences. On the other hand, in the studies by Acosta-Gallego [19], Cedraschi [24], Evcik [26], Fernandes [27], Gusi [31], Sevimli [38], Maindet [36], Tomas-Carus [39, 40] and Latorre Román [33], there were significant results for the pain variable. However, in Fernandes [27] and Sevimli [38], there was a significant improvement between before of aquatic intervention and post-test measures in the experimental group (*p* < 0.001), but this was not present between groups, considering that the comparison group also did aerobic exercise. On the contrary, the Acosta-Gallego [19] and Kurt [32] study showed a significant improvement (*p* = 0.012; *p* < 0.05) between the aquatic therapeutic exercise group and the land aerobic exercise group.

### Effects on psychological outcomes

Depression and anxiety are common symptoms of fibromyalgia. The most relevant instruments used to assess psychological symptoms are the FIQ, Short Form-36 (SF-36), Beck Depression Inventory (BDI) and Hamilton Depression Scale (HAM-D) for depression; Beck Anxiety Inventory (BAI) for anxiety and Fear Avoidance Beliefs Questionnaire (FABQ), Catastrophic Thoughts on Pain Scale (PRCTS) for fear beliefs and catastrophic thoughts and Psychological General Well-Being (PGWB). In the Medeiros study [25], FABQ improved with mat Pilates while PRCTS improved with aquatic exercise, and FIQ showed improvements in both groups. Sevimli [38] also demonstrated significant differences in pre- and post-test in three groups for the BDI, FIQ and mental component of SF-36. Additionally, the Acosta-Gallego [19], Lopez-Rodriguez [34] Tomas-Carus [39, 40] and Kurt [32] study showed considerable improvements in depressive symptoms in both groups (*p* < 0.001; *p* < 0.05). Also, studies such as Assis [22], Cedraschi [24], Gowans [30] and Gusi [31] showed significant differences between groups. In the Andrade [20] study no statistically significant results were obtained regarding depression. However, also in the Andrade [20] study, anxiety was a variable that obtained statistically significant results compared to the control group (*p* < 0.05). The Fonseca [29] study demonstrated improvements in both groups regarding anxiety [*F*(_1.34,58.34_) = 18.98; *p* ≤ 0.01], but no statistically significant results were obtained between groups.

### Effects on physiological outcomes

Patients diagnosed with fibromyalgia also suffer physiological symptoms such as sleep disturbances and fatigue. In the Fonseca [29] study, sleep quality was assessed with the Piper Fatigue Scale-Revised (PFS-R) and demonstrated an effect in both groups. In the Andrade [20] and Acosta-Gallego [19] studies, a time effect for the variable VAS fatigue was observed [*F*(_1.27_) =4.68; *p* = 0.03]. In Maindet [36] study, sleep quality was assessed using PSQI without statistical differences between groups; however, Pichot’s Fatigue Scale showed significant differences between the same groups. In contrast, in the Fonseca [29] study, there were no significant results between groups (*p* > 0.05), despite the fact that significant results were obtained in the post-intervention measure for the control group (*p* ≤ 0.05). On the alternative, the only study that has assessed sleep time is Fernandes de Melo [28] study, where significant changes have been demonstrated that the hydrotherapy group increased at least 1 h of total sleep time regarding conventional physiotherapy (*p* = 0.04).

## Discussion

This systematic review and meta-analysis studied the effectiveness of aquatic therapy on sleep quality, pain, psychological symptoms, quality of life and health status in people diagnosed with fibromyalgia in short, mid and long term. In assessing the risk of bias, none of the studies can be considered displaying a high risk of bias. The bias of blinding participants was considered difficult to follow, especially when delivering an aquatic intervention.

None of the previous reviews on aquatic therapy has specifically analyzed sleep quality. Nevertheless, the systematic review of Bidonde [6] recruited a total of 881 patients from 16 studies, where the interventions were nine studies comparing aquatic exercise versus a control group, five studies comparing aquatic exercise versus land exercise and two studies comparing two different aquatic exercise interventions. Between the water exercise and land exercise groups, no significant differences were obtained except for muscle strength, which was in favour of land exercise. In the studies comparing different aquatic interventions, the only significant difference was for stiffness, favouring the Ai-Chi intervention [44]. The Bidonde [6] study found low- to moderate-quality evidence compared to the control group and suggested that aquatic exercise is beneficial in improving well-being, symptoms and fitness in adults with FM, although there was no significant difference from land-based exercise.

Among the 22 studies, 8 compared aquatic exercise versus a control group [20, 24, 30, 31, 33, 36, 39], 11 compared aquatic exercise versus land exercise [19, 22, 23, 25, 27, 28, 32, 34, 35, 37, 38], one compared aquatic exercise versus FM health education [29] and one compared two different aquatic therapies [21]. Within each study, different measurement variables were assessed, many of which were common, such as FIQ, VAS and SF-36. In the study by Singh et al [45] that explored the sleep disturbances and their impact on quality of life in patients with fibromyalgia, the results suggestsed that 23.5% of variance of FIQR scores were accounted for by PSQI and Autonomic Function Test [45].

In comparing our results with those obtained in the meta-analysis by Lima et al. [8], they included one study [46] comparing aquatic therapy with no treatment while in our meta-analysis all the included studies had an active control group. In this regard, the results from Lima et al. [8] showed statistically significant results in favour or aquatic therapy versus no treatment for sleep quality, when measured with PSQI.

Another noteworthy aspect is the limited precision in the presentation of PSQI data, as none of the studies provided the breakdown of the questionnaire’s various dimensions. Consequently, we are unable to ascertain the specific aspects related to sleep that are improved by aquatic therapy.

In comparing the effectiveness of aquatic therapy versus land exercise, Lima et al. [8] found one study [28] showing aquatic therapy being more effective in improving total sleep time and total nap time, evaluated using a sleep diary. However, in the studies included in the present systematic review, the results showed aquatic biodance being more effective than land stretching for sleep quality measure with PSQI. However, Mat Pilates is equally effective than an aquatic aerobic exercise program for improving sleep quality.

Regarding other interventions, our meta-analysis showed aquatic therapy being equally effective than health education, balneotherapy and balneotherapy combined with exercise in ameliorating sleep quality in a short term, according to PSQI total score results. Aquatic therapy also showed the same benefits than land-based exercises in a short term when evaluated using the FIQ-morning tiredness item. Lima et al. [8] reported that aquatic therapy demonstrated superiority over balneotherapy in the short term, but not in the mid term. Furthermore, aquatic therapy was found to be more effective than recreational activities in the short term, but not as beneficial as Ai Chi in terms of improving sleep quality. Lima et al. [8] also included a study [21] comparing pool versus seawater strength-related benefits. About hydrotherapy or spa therapy, it has shown significant improvement in pain, but with little evidence to suggest the superiority of hydrotherapy over balneotherapy. This corroborates the results presented in our review, in which aquatic exercise did not provide statistically significant different results when compared to land exercise, indicating that one modality is not superior to the other.

For the outcomes in terms of applying aquatic aerobic exercise, it was found that the mechanisms provided by water immersion are directly related to the results. We should also highlight the acute benefits of the temperature of the water can enhance the parasympathetic response and modulate the sympathetic response, which could lead to a reduction in stress-related hormones such as adrenaline, noradrenaline and cortisol and allow the release of serotonin, inducing a momentary improvement in clinical symptoms [47, 48].

Furthermore, an extensive study by Becker [49] on aquatic therapy demonstrated a reduction in pain, improvement in sleep patterns and a positive impact on fibromyalgia and mood-state disorders when compared with land-based exercise programs. The aquatic groups typically showed faster and larger gains, with longer post-study improvements [49]. The findings corroborate our results regarding our long-term FIQ and VAS results that point to aquatic therapy as providing larger gains in the longer follow-up assessments. Besides, there are potential mediators in sleep quality, such as depressive symptoms and mood. A recent review [50] that used mediation analysis found various relationships between pain and sleep quality. Additionally, it determined that sleep quality and pain also were affected by pain attention, pain-related hopelessness, activation of stress in the HPA axis system, fatigue and physical activity. The authors postulate that the psychological and physiological components of emotional experience and attentional processes are likely mediators of the sleep-pain relationship. On the other hand, according to Scott review [51] improving sleep represents a viable treatment target that can confer significant benefits to mental health.

The concept of central sensitization of nociception had been proposed as the primary cause of widespread chronic pain. Clinically, central sensitization is marked by hypersensitivity to mechanical stimuli, while neurophysiologically, it entails substantial enhancements in the membrane excitability and synaptic efficiency of spinal neurons responsible for nociception. It is worth noting that aquatic interventions stimulate different endogenous systems and physiological processes, including the immune, autonomic and endocrine systems and their interactions [48]. Such intervention also influence the presence of neuroinflammation in chronic pain [49].

As with any meta-analysis, there is a potential for selection bias. First, screening references of identified trials may bring positive results because trials with positive results are more likely to be published than trials with negative outcomes. Second, almost all of the included studies were not blinded, and the researchers were not always blinded to the participants’ intervention. Studies involving active patient participation consistently exhibit a bias in blinding due to the inherent interactive nature of such interventions. Consequently, all studies concerning physical activity, physiotherapy or similar interventions are prone to this bias unless comparison is possible with an active control group characterized by null effectiveness. Third, all studies included follow-up in the short or medium term, so the long-term effect of aquatic therapy remains unclear.

## Conclusions

In summary, within the limitations of this study, aquatic therapy as an adjunct treatment to usual care may provide additional benefit in sleep quality for people suffering from fibromyalgia. Aquatic therapeutic exercise improves the symptoms and quality of life of adult patients with fibromyalgia. The current study corroborates the results of previous systematic reviews [6, 8, 52]. Although aquatic therapeutic exercise seems to be as effective as land-based aerobic exercise, there were beneficial outcomes for fibromyalgia achieved in relation to sleep quality, pain, fatigue, quality of life and depression. Further research on long-term outcomes may contribute to the currently available evidence.

### Supplementary information

ESM 1 Funnel plot of comparison: FIQ short term (DOCX 14.9 KB)

ESM 2 Funnel plot of comparison: FIQ mid-term (DOCX 16.0 KB)

ESM 3 Funnel plot of comparison: PSQI (DOCX 16.1 KB)

ESM 4 Funnel plot of comparison: VAS (DOCX 15.2 KB)

ESM 5 Sensitivity analyses of PSQI (DOCX 13.5 KB)

ESM 6 Sensitivity analyses of FIQ short-term (DOCX 12.2 KB)

ESM 7 Sensitivity analyses of FIQ mid-term (DOCX 12.4 KB)

ESM 8 Sensitivity analyses of VAS at mid-term (DOCX 13.5 KB)

## Data Availability

This manuscript has no associated data.
