# Supplementary figures and images for: Aquatic therapy improves self-reported sleep quality in fibromyalgia patients: a systematic review and meta-analysis
Source: Sleep Breath. 2023 Oct 17;28(2):565–83. doi: 10.1007/s11325-023-02933-x (PMC11136798; doi:10.1007/s11325-023-02933-x)

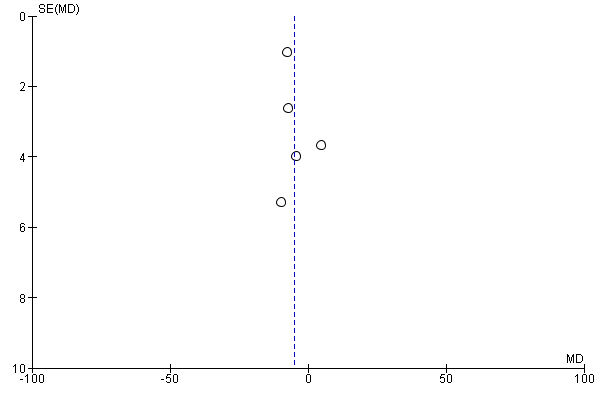

Supplement: Supplementary file 1 — ESM 1 Funnel plot of comparison: FIQ short term (DOCX 14.9 KB) [file 11325_2023_2933_MOESM1_ESM.docx]

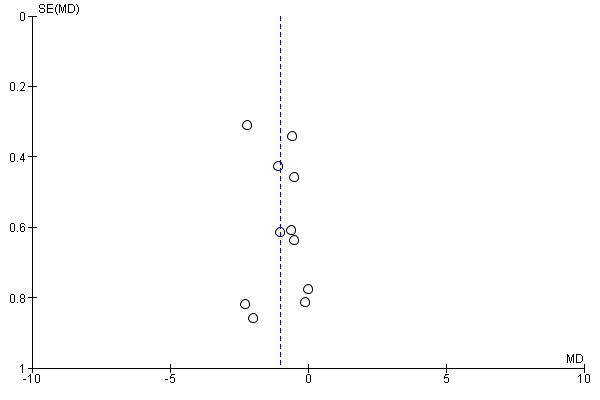

Supplement: Supplementary file 2 — ESM 2 Funnel plot of comparison: FIQ mid-term (DOCX 16.0 KB) [file 11325_2023_2933_MOESM2_ESM.docx]

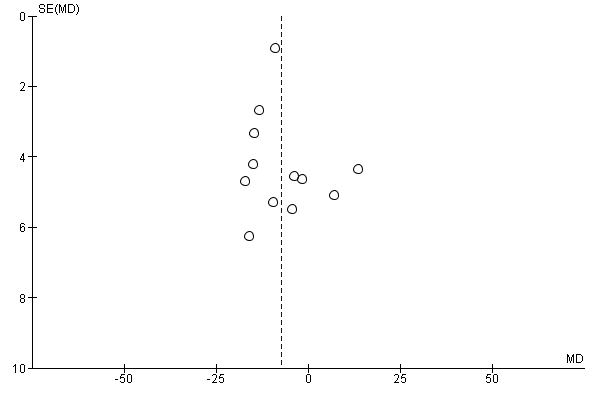

Supplement: Supplementary file 3 — ESM 3 Funnel plot of comparison: PSQI (DOCX 16.1 KB) [file 11325_2023_2933_MOESM3_ESM.docx]

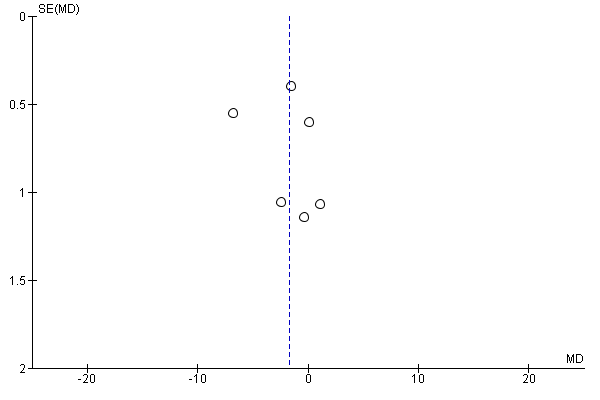

Supplement: Supplementary file 4 — ESM 4 Funnel plot of comparison: VAS (DOCX 15.2 KB) [file 11325_2023_2933_MOESM4_ESM.docx]
