# Supplementary material for: Aquatic therapy improves self-reported sleep quality in fibromyalgia patients: a systematic review and meta-analysis
Source: Sleep Breath. 2023 Oct 17;28(2):565–83. doi: 10.1007/s11325-023-02933-x (PMC11136798; doi:10.1007/s11325-023-02933-x)
Supplement: Supplementary file 5 — ESM 5 Sensitivity analyses of PSQI (DOCX 13.5 KB) [file 11325_2023_2933_MOESM5_ESM.docx]

| Study omitted | Estimate | [95% Conf. | Interval ] |
| --- | --- | --- | --- |
| Andrade, 2019 | 1.09 | -4.41 | 1.26 |
| De Medeiros, 2020 | 1.38 | -4.73 | 0.82 |
| Fonseca, 2019 | 1.64 | -4.93 | 0.43 |
| Kurt, 2016 | 0.98 | -5.16 | 1.72 |
| López-Rodríguez, 2013 | 1.21 | -1.82 | 0.43 |
| Maindet, 2021 | 1.43 | -4.95 | 0.78 |
| Combined | 1.37 | -4.17 | 0.75 |
