# Supplementary material for: Aquatic therapy improves self-reported sleep quality in fibromyalgia patients: a systematic review and meta-analysis
Source: Sleep Breath. 2023 Oct 17;28(2):565–83. doi: 10.1007/s11325-023-02933-x (PMC11136798; doi:10.1007/s11325-023-02933-x)
Supplement: Supplementary file 6 — ESM 6 Sensitivity analyses of FIQ short-term (DOCX 12.2 KB) [file 11325_2023_2933_MOESM6_ESM.docx]

| Study omitted | Estimate | [95% Conf. | Interval ] |
| --- | --- | --- | --- |
| Britto, 2020 | 7.31 | -8.33 | -4.81 |
| Fernandes, 2016 | 7.46 | -8.57 | -5.00 |
| Fonseca, 2019 | 8.06 | -9.16 | -5.57 |
| Kurt, 2016 | 6.98 | -8.42 | -4.73 |
| Maindet, 2021 | 2.23 | -7.60 | -0.66 |
| Combined | 7.51 | -8.39 | -4.92 |
