# Supplementary material for: Aquatic therapy improves self-reported sleep quality in fibromyalgia patients: a systematic review and meta-analysis
Source: Sleep Breath. 2023 Oct 17;28(2):565–83. doi: 10.1007/s11325-023-02933-x (PMC11136798; doi:10.1007/s11325-023-02933-x)
Supplement: Supplementary file 7 — ESM 7 Sensitivity analyses of FIQ mid-term (DOCX 12.4 KB) [file 11325_2023_2933_MOESM7_ESM.docx]

| Study omitted | Estimate | [95% Conf. | Interval ] |
| --- | --- | --- | --- |
| Andrade, 2019 | 2.63 | -11.18 | -1.63 |
| Assis, 2006 | 3.09 | -12.06 | -2.71 |
| Britto, 2020 | 2.91 | -11.70 | -2.27 |
| De Medeiros, 2020 | 3.79 | -12.67 | -4.04 |
| Fernandes, 2016 | 3.10 | -12.20 | -2.75 |
| Fonseca, 2019 | 5.26 | -12.73 | -5.81 |
| Kurt, 2016 | 2.57 | -11.40 | -1.53 |
| López-Rodríguez, 2012 | 2.69 | -10.96 | -1.73 |
| López-Rodríguez, 2013 | 2.70 | -11.19 | -1.77 |
| Maindet, 2021 | 2.28 | -12.80 | -0.96 |
| Sevimli, 2015 | 3.22 | -12.32 | -2.99 |
| Tomas-Carus, 2007 | 2.81 | -11.20 | -1.99 |
| Combined | 3.26 | -11.06 | -2.76 |
