# Supplementary material for: Aquatic therapy improves self-reported sleep quality in fibromyalgia patients: a systematic review and meta-analysis
Source: Sleep Breath. 2023 Oct 17;28(2):565–83. doi: 10.1007/s11325-023-02933-x (PMC11136798; doi:10.1007/s11325-023-02933-x)
Supplement: Supplementary file 8 — ESM 8 Sensitivity analyses of VAS at mid-term (DOCX 13.5 KB) [file 11325_2023_2933_MOESM8_ESM.docx]

| Study omitted | Estimate | [95% Conf. | Interval ] |
| --- | --- | --- | --- |
| Acosta-Gallego, 2018 | 3.80 | -1.61 | -0.52 |
| Andrade, 2019 | 3.54 | -1.56 | -0.45 |
| Assis, 2006 | 4.06 | -1.60 | -0.56 |
| Britto, 2020 | 3.98 | -1.59 | -0.54 |
| De Medeiros, 2020 | 3.73 | -1.59 | -0.49 |
| Evcik, 2008 | 3.35 | -1.56 | -0.48 |
| Fernandes, 2016 | 3.79 | -1.59 | -0.51 |
| Latorre Román, 2015 | 3.48 | -1.44 | -0.40 |
| Maindet, 2021 | 3.72 | -1.62 | -0.50 |
| Sevimli, 2015 | 4.43 | -1.12 | -0.43 |
| Tomas-Carus, 2007 | 3.49 | -1.47 | -0.41 |
| Combined | 3.38 | -1.51 | -0.50 |
